# Up-regulating autophagy by targeting the mTOR-4EBP1 pathway: a possible mechanism for improving cardiac function in mice with experimental dilated cardiomyopathy

**DOI:** 10.1186/s12872-020-01365-9

**Published:** 2020-02-04

**Authors:** Bo Jin, Haiming Shi, Jun Zhu, Bangwei Wu, Quzhen Geshang

**Affiliations:** 1 Department of Cardiology, Huashan Hospital, Fudan University, 12 Middle Urumqi Road, Shanghai, 200040 China; 2 Department of Medicine, Medical College of Tibet University, Lasa, Tibet China

**Keywords:** Autophagy, mTOR, Cardiac function, Dilated cardiomyopathy

## Abstract

**Background:**

Autophagy plays a crucial role in the pathological process of cardiovascular diseases. However, little is known about the pathological mechanism underlying autophagy regulation in dilated cardiomyopathy (DCM).

**Methods:**

We explored whether up-regulating autophagy could improve cardiac function in mice with experimental DCM through the mTOR-4EBP1 pathway. Animal model of DCM was established in BALB/c mice by immunization with porcine cardiac myosin. Both up- or down-regulation of autophagy were studied by administration of rapamycin or 3-MA in parallel. Morphology, Western blotting, and echocardiography were applied to confirm the pathological mechanisms.

**Results:**

Autophagy was activated and autophagosomes were significantly increased in the rapamycin group. The collagen volume fraction (CVF) was decreased in the rapamycin group compared with the DCM group (9.21 ± 0.82% vs 14.38 ± 1.24%, *P* < 0.01). The expression of p-mTOR and p-4EBP1 were significantly decreased in rapamycin-induced autophagy activation, while the levels were increased by down-regulating autophagy with 3-MA. In the rapamycin group, the LVEF and FS were significantly increased compared with the DCM group (54.12 ± 6.48% vs 45.29 ± 6.68%, *P* < 0.01; 26.89 ± 4.04% vs 22.17 ± 2.82%, *P* < 0.05). As the inhibitor of autophagy, 3-MA aggravated the progress of maladaptive cardiac remodeling and declined cardiac function in DCM mice.

**Conclusions:**

The study indicated a possible mechanism for improving cardiac function in mice with experimental DCM by up-regulating autophagy via the mTOR-4EBP1 pathway, which could be a promising therapeutic strategy for DCM.

## Background

Dilated cardiomyopathy (DCM) is one of the most common cardiomyopathy worldwide characterized by left ventricular dilation and decline in contraction function, which is the third leading cause of congestive heart failure [1–3]. Recent studies of pathological mechanisms underlying heart failure focus on structural changes in cardiomyocytes and extracellular matrix to explain the deleterious contractile function [4, 5]. However, the exact molecular mechanism in the onset and progression of DCM is still unclear.

Cardiovascular diseases including hypertrophic and ischemic cardiomyopathies are increasingly being reported to accumulate misfolded proteins and damaged organelles. As the highly conserved pathway, autophagy plays a crucial role in the pathological process of cardiovascular diseases. Previous studies indicated that autophagy is activated in maladaptive cardiac remodeling of chronic heart failure [6–8]. However, little is known about the pathological mechanism underlying autophagy regulation by pharmacological interventions in dilated cardiomyopathy. There are still many unanswered questions and points of confusion that have yet to be resolved. Therefore, an in-depth investigation into the molecular mechanism is vital to therapeutic interventions in the field. Here, we focuses on whether regulating autophagy could improve cardiac function in DCM mice through the mTOR-4EBP1 pathway.

To the best of our knowledge, little information is available to confirm whether up-regulating autophagy could improve cardiac function in DCM mice. In the present study, we propose that autophagy regulation plays a crucial role in determining cardiac function in mice with experimental DCM through the mTOR-4EBP1 pathway. Therefore, we pinpointed that up-regulating autophagy by targeting the mTOR-4EBP1 pathway as the possible molecular mechanisms in cardio-protection in DCM mice. Both up- or down-regulation of autophagy were investigated by administration of rapamycin or 3-methyadenine (3-MA) in parallel. Based on these findings, modulating autophagy could be a potential therapeutic target to minimize myocardial injury and optimize the restoration of cardiac function [9–11].

## Methods

### Animal models and experimental design

All animal experiments were approved by the Animal Care and Utilization Committee of Fudan University (201802021S). We obtained the male BALB/c mice aged 6 weeks from Fudan University Experimental Animal Center. The animal model of DCM was established in BALB/c mice by injection with porcine cardiac myosin (Sigma). Cardiac myosin was emulsified with an equal volume of complete Freund’s adjuvant (Sigma) to the concentration of 2 mg/ml. The cardiac myosin was subcutaneously injected into the groin of BALB/c mice twice at days 0 and day 7. The total dose for DCM induction was 0.2 mg per mouse. The mice in the control group were injected with complete Freund’s adjuvant as the vehicle. As previously reported [12], we confirmed myosin-induced DCM model by histomorphological study and echocardiographic assessments in the present study. Additional eight normal mice and twenty-four DCM mice were divided into the following four experimental groups as follows: control group (normal + PBS), DCM group (DCM + PBS), rapamycin group (DCM + rapamycin) and 3-MA group (DCM + 3-MA). Eight weeks after immunization, rapamycin was then administered at a dose of 2 mg/kg/d for 2 weeks. The mice in 3-MA group received 3-MA at a dose of 15 mg/kg/d, while the mice in the control group were injected with PBS alone. After intraperitoneal injection of sodium pentobarbital (75 mg/kg body weight), all mice were sacrificed by cervical dislocation while anesthetized.

### Echocardiographic measurements

M-mode transthoracic echocardiography was performed using a 30-MHz imaging transducer to evaluate the cardiac function. The mice in the four experimental groups were anesthetized with 2% isoflurane and their chests were epilated. M-mode images were obtained at the level of papillary muscles in the long-axis view. The left ventricular ejection fraction (LVEF), fractional shortening (FS), left ventricular end-diastolic dimension (LVEDD), and left ventricular end-diastolic volume (LVEDV) were measured, which were acquired by the technician who was blinded to the present experimental groups.

### Histopathology image analysis

Myocardial tissues were obtained and fixed in 4% formaldehyde, embedded in paraffin and cut into 5 μm thick slices. Specimens were treated and stained with picrosirius red, and microscopic images were observed. The collagen volume fraction (CVF) was measured by quantitative morphometry of specimens with IMS Cell Image Analysis System (Shen Teng, Shanghai, China). Morphological changes were investigated under confocal scanning microscope (Leica, TCS-SP2, Germany). For quantitation of cardiac fibrosis areas, 5 random fields of view per mouse were evaluated for CVF analysis across the left ventricular section (Each group, *n* = 8). Consequently, there were 40 quantitative data for statistical analysis in each group.

### Transmission electron microscopy evaluation

Transmission electron microscopy (TEM) for morphological evaluation was performed at Electron Microscopy Core Laboratory, Shanghai medical college, Fudan University (Philips CM120, Nethelands), according to standard operating procedures. As previously reported for morphological TEM [13], cardiac tissues were fixed in 2.5% glutaraldehyde in phosphate buffer overnight at 4 °C. After sample preparation, 90-100 nm thick sections were mounted onto a 200 mesh copper grid and examined under a Philips CM120 electron microscope. The pathological alterations of cardiomyocyte nucleus, mitochondria, myocardial fibers, and autophagosomes were evaluated in the four groups.

### Western blotting assays

After being harvested, the left ventricular myocardium specimens were stored at − 80 °C. Proteins were extracted from the myocardial tissues homogenized in RIPA Lysis (Beyotime) and Extraction Buffer with a protease inhibitor cocktail, and proteins were quantified using the bicinchoninic acid method according to the manufacturer’s instructions. The total of 25 μg protein samples were loaded into 8% SDS-PAGE gels for electrophoresis then transferred to PVDF membranes over night at 30 V. Antibodies specific for LC3 II (dilution 1:1000; Cell Signaling), p-mTOR (dilution 1:1000; Cell Signaling), and p-4EBP1 (dilution 1:1000; Cell Signaling) were incubated at 4 °C overnight, and GAPDH (dilution 1:5000; Santa Cruz) was used as a loading control to normalize gel loading and protein expression. HRP-conjugated secondary antibodies plus ECL were incubated at 37 °C for 1 h for protein visualization. The densitometric values of immunoreactive bands were measured using Image J (NIH, USA).

### Statistical analysis

The data are presented as mean ± standard deviation. Values of *P* less than 0.05 were considered statistically significant. Normal distribution was confirmed in four experimental groups, and differences in means between two groups were analyzed by unpaired Student’s t test when the data were normally distributed. Multiple group comparison was performed by one-way ANOVA followed by Newman-Keuls multiple comparison test. GraphPad Prism version 6.0 software (GraphPad Software Inc., USA) was used for data analysis.

## Results

### General characteristics

The animal model was successfully established in male BALB/c mice, and twenty-four DCM mice were randomly divided into DCM group, rapamycin group, and 3-MA group equally. Furthermore, eight normal mice in the control group were administered with Freund’s adjuvant alone. No significant difference was found in the body weight, heart weight and heart weight/body weight (HW/BW), although a tendency was found that the body weight was slightly decreased in the 3-MA group, it did not reach the statistically significant level (Table 1).

**Table 1:** The general characteristics of the four experimental groups

	Control group	DCM group	Rapamycin group	3-MA group
Number of death	0	1	0	0
Body weight (g)	19.87 ± 2.40	19.72 ± 2.22	19.69 ± 2.16	19.59 ± 2.27
Heart weight (g)	0.22 ± 0.02	0.21 ± 0.01	0.22 ± 0.02	0.23 ± 0.03
HW/ BW (mg/g)	11.09 ± 1.25	10.94 ± 1.08	11.15 ± 1.21	11.53 ± 1.33

*HW/ BW* Heart weight/ Body weight (mg/g); Each group, *n* = 8

### Modulating autophagy and morphological evaluation

**Fig. 1:**
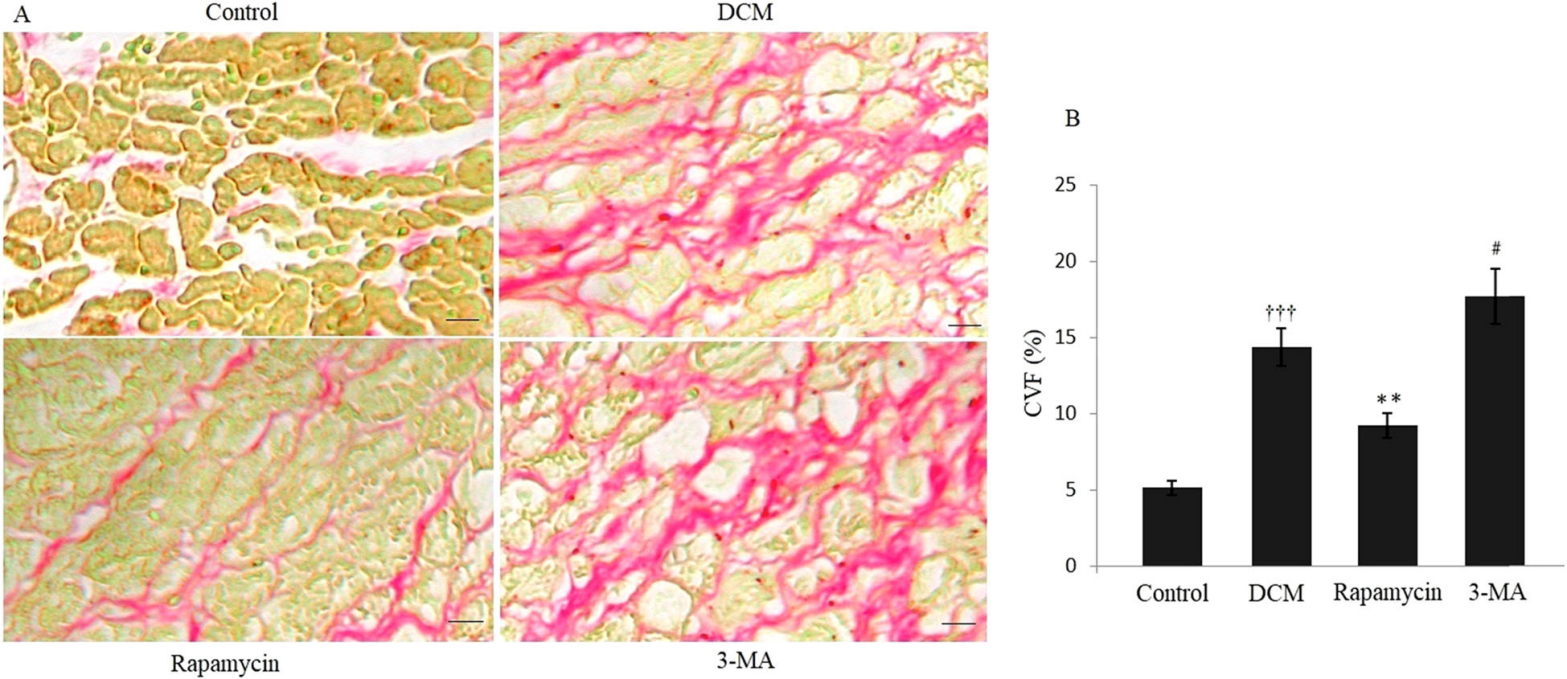
Modulating autophagy and cardiac matrix remodeling of DCM. (A) Picrosirius red staining indicated significantly changes of collagen distribution in the four different groups. (B) Histochemical analysis showed that there was a significant increase of collagen distribution in the DCM group compared with the control group. Quantitative assessment demonstrated that the CVF was significantly decreased in the rapamycin group, and it was increased in the 3-MA group compared with the DCM group. ^†††^*P* < 0.001 vs Control, ^**^P < 0.01 and ^#^*P* < 0.05 vs DCM. Scale bar = 100 μm

The experimental model of DCM was established in BALB/c mice by immunization with porcine cardiac myosin. Histochemical analysis with picrosirius red staining indicated that there was a significant increase of CVF in the DCM group compared with the control group, revealing cardiac fibrosis in DCM mice. Figure 1 indicated that the CVF was significantly decreased in the rapamycin group than the DCM group (9.21 ± 0.82% vs 14.38 ± 1.24%, *P* < 0.01). However, the CVF was increased to 17.68 ± 1.81% by down-regulating autophagy in the 3-MA group compared with the DCM group (*P* < 0.05).

**Fig. 2:**
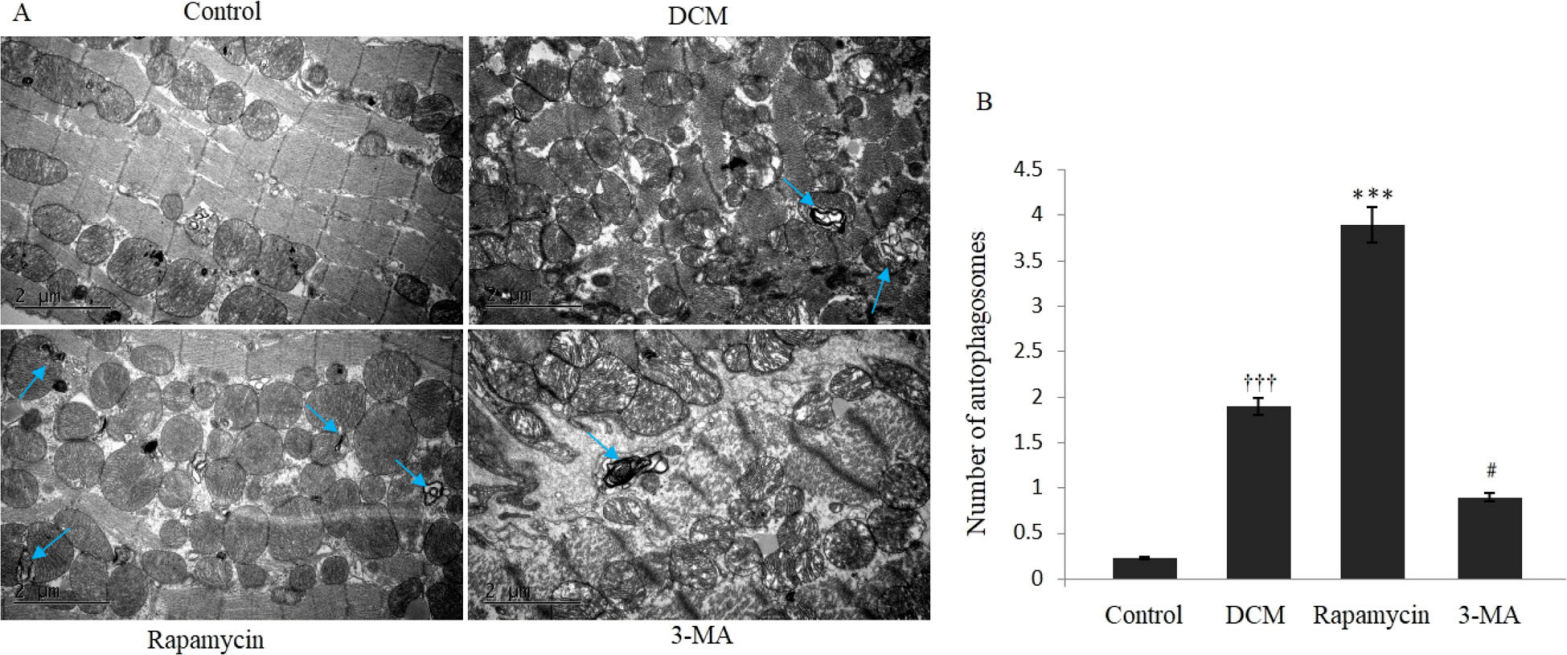
Transmission electron microscopy assessment for modulating autophagy. (A) Transmission electron microscopy indicated significant changes of autophagosomes in the four different groups. (B) Transmission electron microscopy showed that there was a significant increase of autophagosomes in the DCM group compared with the control group. Quantitative assessment demonstrated that autophagosomes were significantly increased in the rapamycin group, and they were decreased in the 3-MA group compared with the DCM group. ^†††^*P* < 0.001 vs Control, ^***^P < 0.01 and ^#^*P* < 0.05 vs DCM. The arrows indicated the double membrane autophagosomes in the different groups

For morphological TEM, normally arranged myofibrils within the sarcomeres with defined Z-bands were observed in the control group. Autophagy was significantly activated and autophagosomes could be confirmed in mice with experimental DCM, and sarcomeric disarray and myofibrillar lysis could be observed. As shown in Fig. 2, double membrane autophagosomes were significantly increased in the rapamycin group compared with the DCM group (*P* < 0.001). We inhibited the autophagy activation by 3-MA and verified that the number of autophagosomes was statistically decreased compared with the DCM group, and the sarcomeric disarray failed to get reversed.

### Modulating autophagy and mTOR-4EBP1 pathway

**Fig. 3:**
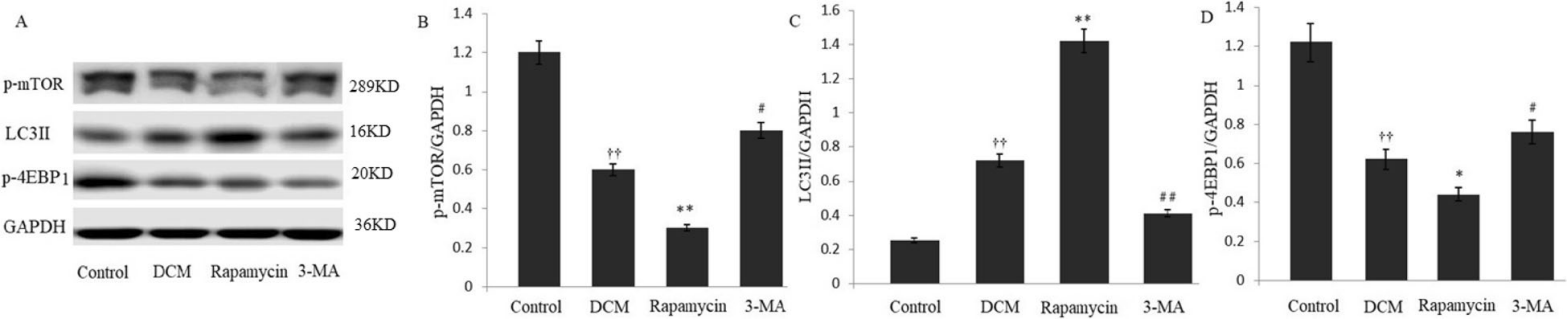
Modulating autophagy and the mTOR-4EBP1 pathway. **a**-**d** The expression levels of p-mTOR and p-4EBP1 were significantly decreased in rapamycin-induced autophagy activation, and the effects were significantly increased by down-regulating autophagy with 3-MA. The increased expression of LC3 II indicated the activation of autophagy in the rapamycin group, whereas the expression of LC3 II was decreased in the 3-MA group. ^††^*P* < 0.01 vs Control, ^**^P < 0.01, ^*^P < 0.05, ^##^*P* < 0.01, and ^#^*P* < 0.05 vs DCM. Each experiment was conducted 3 times in triplicate

The conversion of LC3 I to LC3 II form is recognized as indicators of autophagy activation. To validate the relationship of autophagy and mTOR-4EBP1 pathway, the p-mTOR and the downstream molecule of p-4EBP1 were measured. Autophagy and mTOR-4EBP1 pathway were regulated in mice with experimental DCM by administration of rapamycin or 3-MA in parallel. Our study indicated that rapamycin-induced inhibition of mTOR-4EBP1 pathway, shown as decreased p-mTOR and p-4EBP1 expression compared with the DCM group. The increased expression of LC3 II indicated the activation of autophagy in the rapamycin group. With the administration of 3-MA, protein levels of p-mTOR and p-4EBP1 were significantly increased, whereas the expression of LC3 II was decreased in the 3-MA group (Fig. 3).

### Modulating autophagy and cardiac function

**Fig. 4:**
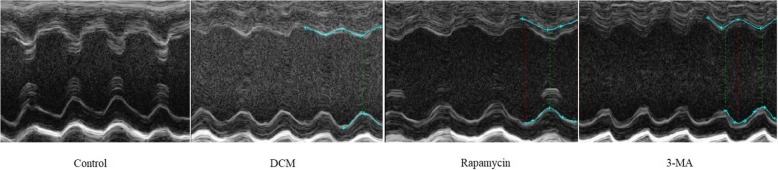
M-mode ultrasound images at the level of papillary muscles in the long-axis view. The M-mode echocardiography evaluation included the following four experimental groups as follows: control group, DCM group, rapamycin group, and 3-MA group. The red lines and blue lines indicated the LVEDD and LVESD respectively. Each group, *n* = 8

**Fig. 5:**
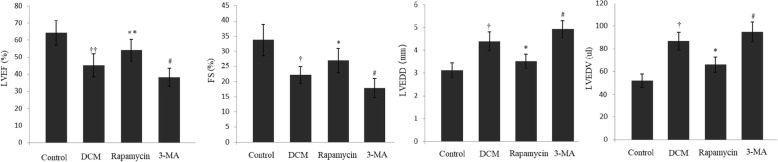
Modulating autophagy and cardiac function. (A) and (B) Cardiac function differed in the four groups, and the LVEF and FS significantly improved in the rapamycin group compared with the DCM group. (C) and (D) The LVEDD and LVEDV were significantly decreased in the 3-MA group compared with the DCM group. ^††^*P* < 0.01 and ^†^*P* < 0.05 vs Control, ^**^P < 0.01, ^*^P < 0.05, and ^#^*P* < 0.05 vs DCM. Each group, *n* = 8

We next examined whether the cardiac function was improved in mice with experimental DCM by up-regulating autophagy. Therefore, M-mode images were obtained at the level of papillary muscles in the long-axis view (Fig. 4). As summarized in Fig. 5, cardiac function differed significantly among the four groups. In the DCM group, the LVEF and FS significantly deteriorated compared with the control group. In the rapamycin group, the parameters were significantly increased compared with the DCM group (54.12 ± 6.48% vs 45.29 ± 6.68%, *P* < 0.01; 26.89 ± 4.04% vs 22.17 ± 2.82%, *P* < 0.05), although cardiac function was still lower than the control group. Furthermore, the LVEDD and LVEDV significantly decreased following down-regulation of mTOR-4EBP1 pathway to activate autophagy. However, 3-MA-induced inhibition of autophagy provided a negative effect to promote the maladaptive cardiac remodeling, possibly in part, by up-regulation of mTOR-4EBP1 pathway involved in the pathological process of DCM.

## Discussion

Experimental autoimmune myocarditis in mice is a typical animal model which mimics the pathophysiological process of DCM. For the first time we reported the cardio-protective effects of rapamycin-induced autophagy activation, which contributed to improve cardiac function in DCM mice via regulating the mTOR-4EBP1 pathway. The biological effects were tested in experimental DCM mice by administration of rapamycin or 3-MA respectively. We confirmed that autophagy was directly activated by down-regulating the mTOR-4EBP1 pathway, which increased the expression of LC3 II and the formation of autophagosomes. Our study indicated that up-regulating autophagy could be a promising therapeutic strategy to improve cardiac function for the pathological progression of DCM.

Autophagy is a highly conserved cellular recycling process, which not only plays an important role in cellular homeostasis but also participates in physiological processes [14, 15]. Autophagy degrades the recycling material in the cell while the former makes it through the formation of double-membrane vesicles that fuse with the lysosomal [16–18]. It plays the major role of catabolic mechanism degenerating and recycling long-lived protein and organelles involving in physiological and pathological process. Accordingly, dysfunction of this process contributes to the pathological process of cardiovascular diseases.

The mTOR pathway is a well-known negative regulator of autophagic activity, which has been established to regulate cell growth, proliferation, and metabolism [19–21]. Our previous study indicated that autophagic activity was up-regulated in a rat model of early-stage dilated cardiomyopathy, which was a part of the reparative processes during DCM progression [22]. As a mTOR inhibitor, rapamycin can dephosphorylate the downstream effectors such as 4EBP1. Furthermore, 4EBP1 is a translation regulator, its dephosphorylation by mTOR inhibitors suppresses overall cellular protein synthesis and induces autophagy [23]. In our present study, rapamycin-induced autophagy activation successfully reversed myocardial fibrosis and improved cardiac function in DCM mice. In contrast, down-regulating autophagy inhibited the formation of autophagosomes in the 3-MA group, which induced severe myocardial fibrosis and decreased cardiac function.

Echocardiography showed that the LVEF and FS significantly decreased in the DCM group, which is consisted with the pathological development of DCM. With the administration of 3-MA, cardiac function failed to improve compared with the DCM group. In the rapamycin group, cardiac function significantly improved compared with the DCM group, although the LVEF and FS were still lower compared with the control group. Meanwhile, the LVEDD and LVEDV statistically reduced by down-regulation of mTOR-4EBP1 pathway to activate autophagy.

The molecular mechanisms of autophagy regulation remain unclear, an in-depth study of mTOR-4EBP1 pathway might thus contribute to provide an exciting therapeutic strategy for DCM [24–26]. Our data indicated that rapamycin down-regulated the mTOR-4EBP1 signaling pathway in DCM mice. To confirm the effect of autophagy inhibition, 3-MA was employed into the present study, which decreased the formation of autophagosomes and activated the pathway as indicated by increase of p-mTOR and p-4EBP1 expression. Data in all demonstrated that directly targeting on the mTOR-4EBP1 pathway was a possible mechanism in the regulation of autophagy in DCM.

Our study was carefully designed and conducted in animal experimental center of Fudan University. Both up- or down-regulation of autophagy were studied by administration of rapamycin or 3-MA in parallel. Some limitations of this study should be acknowledged. We preliminary explored the mechanisms for improving cardiac function induced partially by regulating the mTOR-4EBP1 pathway, so other aspects of critical molecular mechanisms should be focused in the future research and is needed to develop better pharmacological interventions. Through further research, a more complete picture of the molecular mechanism and regulation of autophagy will strengthen our understanding of the pathological process.

## Conclusions

The present study indicated that autophagy activation was involved in the pathological progress of experimental DCM. As a possible molecular mechanism, up-regulating autophagy contributed to improve cardiac function in part through mTOR-4EBP1 pathway, which could be a promising therapeutic strategy for DCM.

## Data Availability

All relevant data is presented in the manuscript and supporting materials. The datasets used and analysed during the current study are available from the corresponding author on reasonable request.

## Abbreviations

3-MA: 3-methyadenine
CVF: Collagen volume fraction;
DCM: Dilated cardiomyopathy
FS: Fractional shortening
LVEDD: Left ventricular end-diastolic dimension
LVEDV: Left ventricular end-diastolic volume
LVEF: Left ventricular ejection fraction
LVESD: Left ventricular end-systolic dimension
SD: Standard deviation
TEM: Transmission electron microscopy
